# Improvement of quality of life and survival using self-expandable metal stent placement for severe malignant stenosis of the gastric body: a case report

**DOI:** 10.1186/1752-1947-6-315

**Published:** 2012-09-19

**Authors:** Hozumi Kumagai, Kenta Nio, Tsuyoshi Shirakawa, Keita Uchino, Hitoshi Kusaba, Taichi Isobe, Masato Komoda, Shingo Tamura, Ryo Maeyama, Eishi Nagai, Koichi Akashi, Eishi Baba

**Affiliations:** 1 Department of Hematology and Oncology, Kyushu University Hospital, 3-1-1 Maidashi, Higashi-ku, Fukuoka 812-8582, Japan; 2 Department of Medical Oncology, Kyushu Medical Center, 1-8-1 Jigyohama, Chuo-ku, Fukuoka, 810-0065, Japan; 3 Department of Surgery and Oncology, Kyushu University Hospital, 3-1-1 Maidashi, Higashi-ku, Fukuoka, 812-8582, Japan

## Abstract

**Introduction:**

Advanced gastric carcinoma often decreases quality of life because of upper gastrointestinal tract stenosis. Self-expandable metal stents have been thought to be an effective, minimally invasive treatment for stenosis. However, the effectiveness of self-expandable metal stent placement for carcinomatous stenosis of the gastric body and antrum has not been clarified, and there have been few reports of such cases.

**Case presentation:**

A 74-year-old Japanese woman developed stenosis of the gastric body and antrum caused by advanced gastric cancer during first-line chemotherapy. She developed weight loss and poor nutrition due to inadequate intake. Self-expandable metal stent placement for stenosis of the gastric body and antrum ameliorated her symptoms rapidly and improved her general condition and quality of life. Eight days after self-expandable metal stent placement, second-line chemotherapy could be administered safely. Oral intake and nutritional status were maintained for 117 days after self-expandable metal stent placement, and she died of gastric cancer 176 days after self-expandable metal stent placement and initiation of second-line chemotherapy.

**Conclusions:**

Self-expandable metal stent placement for carcinomatous stenosis in the gastric body and antrum could be an effective therapeutic strategy for patients with inadequate oral uptake. It may provide rapid improvement of the patient’s general condition and oral intake with minimal complications, comparatively long-term symptom relief, and a survival benefit by allowing second-line chemotherapy.

## Introduction

Advanced gastric carcinoma is a serious disease that not only has a poor prognosis but also decreases quality of life (QOL) because of upper gastrointestinal tract stenosis. Bypass surgery has been conventionally performed to relieve this condition and improve the QOL of these patients [1]. Although palliative gastrostomy and ileostomy and gastric tube insertion have been performed for patients in poor general condition previously [2,3] (many of whom could not undergo bypass surgery), self-expandable metal stents (SEMS) have been recognized as an effective, minimally invasive treatment in recent years [4-6]. SEMS are made of plastic materials and may be used to treat patients with malignant obstruction or external compression of the upper gastrointestinal tract, malignant gastrointestinal perforations, and selected cases of benign upper gastrointestinal disease. However, the effectiveness of SEMS placement for gastric carcinomatous stenosis with diffuse extensive cancer cell infiltration is unclear because there have been few reports of such cases. A case in which SEMS placement followed by second-line chemotherapy ameliorated gastric carcinomatous stenosis, gave comparatively long-term relief, and improved QOL is described here. The usefulness, safety, and problems of this treatment are discussed.

## Case presentation

**Figure 1:**
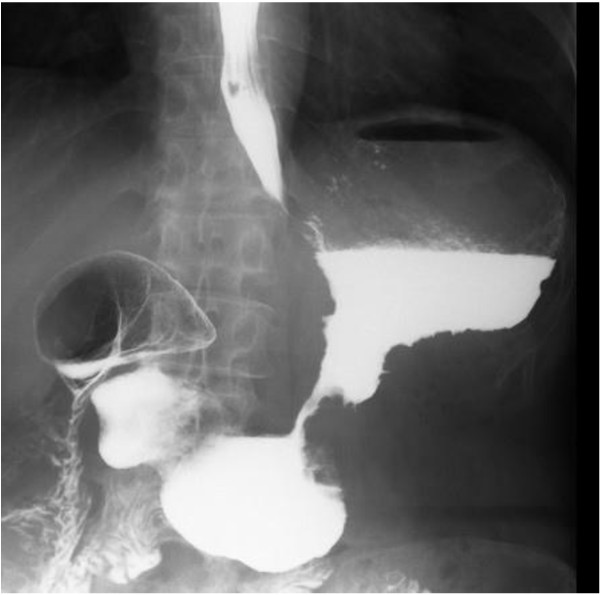
**Contrast radiography of our patient on their initial visit.** Scirrhous type gastric cancer was distributed in the wide area of the stomach with stricture of the gastric body seen on contrast radiography.

**Figure 2:**
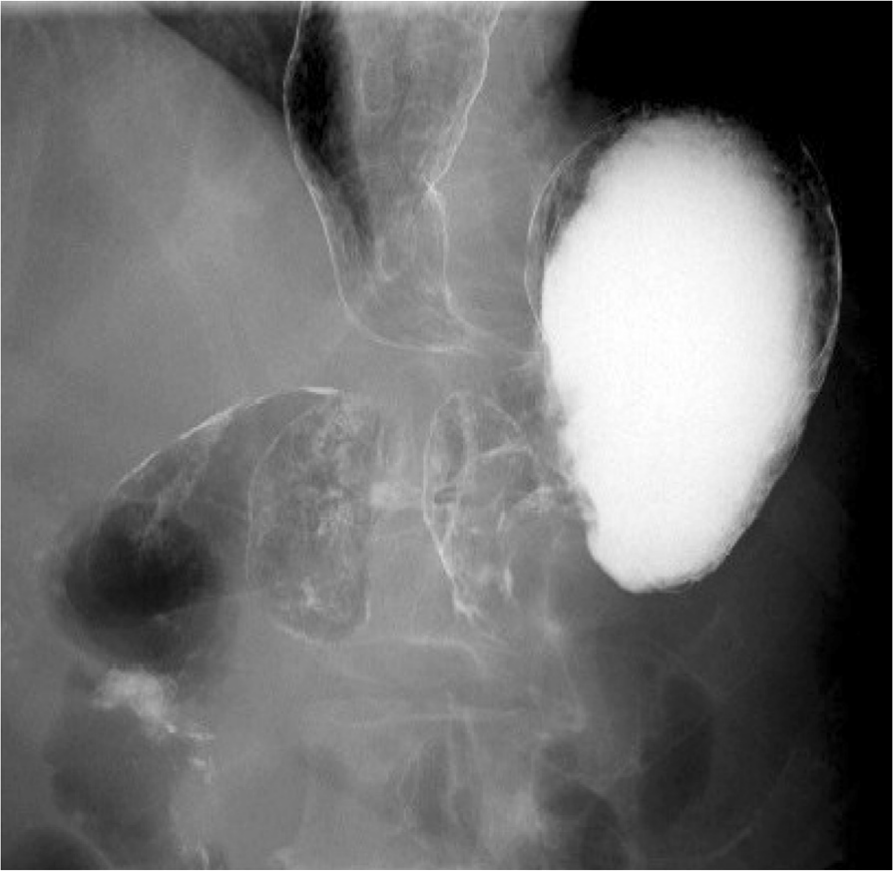
**Contrast radiography after the fourth cycle of first-line chemotherapy.** Contrast radiography revealed two sections of stenosis in the esophago-gastric junction and gastric body. The length of the stenosis from the gastric lower body to the antrum was 4cm, and the pyloric ring was intact.

A 74-year-old Japanese woman visited her local clinic complaining of tarry stool one month prior to visiting our department. She was referred to our department for further examination. Contrast radiography and endoscopy of the upper gastrointestinal tract revealed a scirrhous type gastric cancer located in a wide area of the stomach infiltrating the esophagogastric junction (EGJ), gastric body, and antrum, with partial ulceration (Figure 1). Biopsy specimens obtained from that lesion showed a poorly-differentiated adenocarcinoma with signet-ring cells. Immunohistochemically, the adenocarcinoma cells were negative for human epidermal growth factor receptor 2 (HER2; score 0). At this point, passage of contrast agent through the stomach was possible. Whole body computed tomography (CT) scans revealed multiple metastases to the abdominal lymph nodes, liver, and peritoneum. A moderate amount of malignant ascites were also found. She had no relevant personal or family history. Her laboratory data showed no major organ dysfunction except for a slight microcytic hypochromic anemia. Her serum levels of carcinoembryonic antigen (CEA) were within normal limits, but carbohydrate antigen (CA) 19-9 levels were elevated to 50.0U/mL (normal range of CA19-9: 0.0 to 37.0U/mL). Her Eastern Cooperative Oncology Group (ECOG) performance status (PS) was 1. PS1 is defined as restricted in physically strenuous activity but ambulatory and able to carry out work of a light or sedentary nature. Since curative surgery was thought impossible, our patient was treated with systemic chemotherapy consisting of cisplatin and S-1, an oral dihydropyrimidine dehydrogenase (DPD)-inhibiting fluoropyrimidine; 60mg/m^2^ of cisplatin was infused over a two-hour period on day one, and 120mg/body/day of S-1 was orally administered on days one to 14 every three weeks. Two months after the initial course of chemotherapy, disappearance of the metastatic liver tumors and improvement of the malignant ascites were observed. Her serum CA19-9 levels had also decreased to within normal ranges. However, gradual appetite loss had appeared during the fourth cycle in March 2011. Upper gastrointestinal endoscopic examination revealed two sections of stenosis in the EGJ and gastric body, showing progression of disease. A large amount of undigested food residue remained, resulting in a gastric outlet obstruction scoring system (GOOSS) score of 1 (score 0: no oral intake; score 1: liquids only; score 2: soft foods; score 3: solid foods/full diet) [1]. Contrast radiography revealed that the length of the stenosis from the gastric lower body to the antrum was 4cm, and, fortunately, the pyloric ring was intact (Figure 2). CT scans showed that the metastatic lesions had not deteriorated, but PS2 is defined as ambulatory and capable of all selfcare but unable to carry out any work activities. Up and about more than 50% of waking hours, and the ascites increased. Gastro-jejunal bypass surgery was not thought possible due to infiltration of carcinomatous cells across a wide area of the stomach. It was finally decided to perform SEMS placement to achieve early symptom relief based on the following reasons: limited stenosis involving the esophago-gastric junction and gastric antrum, life expectancy of more than several months, and our patient’s wishes. 

**Figure 3:**
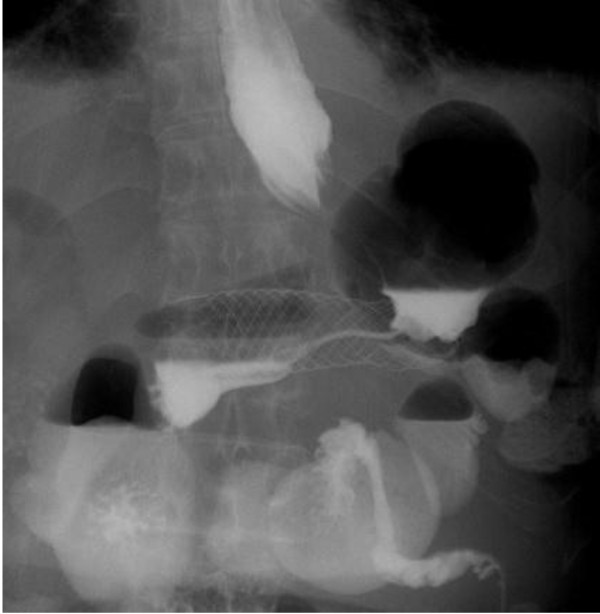
**Contrast radiography on the day after stent placement.** The self-expandable metal stent was fully expanded and the carcinomatous stricture of the gastric body was released, as seen on contrast radiography.

**Figure 4:**
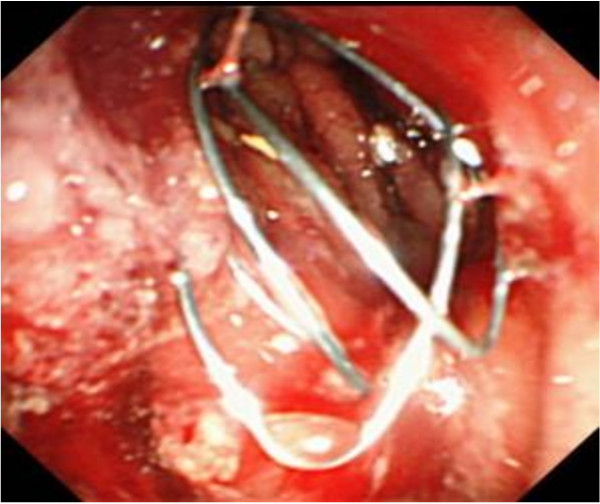
**Endoscopic image of the self-expandable metal stent.** Stent placement at the stenotic lesion of the gastric body was successfully performed.

SEMS placement was performed targeting the gastric antral stenosis on April 2011 using the Olympus® Model GIF-XP260 and GIF-XQ240 (Olympus, Tokyo, Japan). After balloon dilatation of the stenotic area, the delivery catheter was inserted from the oral side of the stenosis along a pre-placed guide wire, and then the SEMS (6cm × 22mm, WallFlex® Duodenal Stent, Boston Scientific, Boston, MA, USA) was placed under radiographic guidance (Figure 3). A small amount of bleeding from the carcinomatous mucosa and edematous change of the EGJ from friction with the endoscope occurred (Figure 4). No severe adverse events were observed during the SEMS placement procedures. For seven days after SEMS placement, our patient’s performance status and nutritional condition deteriorated because of worsening of dysphagia induced by the edematous change of the EGJ (GOOSS score 0). Along with disappearance of the edema eight days after SEMS placement, the dysphagia improved enough to allow oral ingestion. Eight days after SEMS placement, 80mg/m^2^ of paclitaxel (PTX) could be safely administered as second-line chemotherapy (SLC). PTX was administered on days one, eight, and 15 every four weeks. Oral ingestion and nutritional status were restored (GOOSS score 3, PS1) after starting SLC. These conditions were maintained for 117 days until EGJ obstruction occurred due to disease progression. The major adverse events of the chemotherapy were only mild general fatigue (Common Terminology Criteria for Adverse Events [CTCAE] version 4.0 grade 1) and minimal lasting peripheral neuropathy (grade 1). Our patient died of cancerous cachexia resulting from disease progression 176 days after SEMS placement and initiation of SLC.

## Discussion

Our patient’s case showed progression of stenosis of the gastric body and antrum along with disease progression during first-line chemotherapy. Prompt amelioration of symptoms and improvement of her general condition were needed because our patient had developed weight loss and poor nutritional status due to inadequate oral uptake. There are several therapeutic options to facilitate oral uptake for patients with stenosis of the upper gastrointestinal tract, including chemotherapy, bypass surgery, and stent placement [5,6]. Insertion of a nasogastric tube and gastrostomy enable only symptom relief by reducing pressure in the gastrointestinal tract [2,3]. Although it has been estimated that SLC induces tumor response in 20% of cases, it might not permit immediate symptom relief [7-9]. In our patient’s case, bypass surgery was not possible because the infiltrating carcinomatous cells were distributed across a wide area of the stomach. We then considered stent placement in the upper gastrointestinal tract. A previous report proposed the indications and contraindications of SEMS for gastrointestinal tract stenosis [4]. The indications were inoperable malignant gastro-duodenal outlet obstruction, extrinsic compression by neoplastic or nodal disease, anastomotic tumor recurrence after surgery, malignant fistula to adjacent organs, benign strictures refractory to balloon dilatation, and patients not being amenable to surgery. The contraindications were curable disease by multimodality treatment, uncorrectable coagulopathy, terminally ill patients with limited life expectancy, peritoneal carcinomatosis with distal small bowel obstruction, free gastrointestinal perforation, bowel ischemia, and sepsis. However, favorable sites for stenoses in the upper gastrointestinal tract for SEMS were not well defined, and there have been few reports of SEMS placement for wide stenotic lesions of the gastric body and antrum because SEMS had not been thought suitable for such cases [10,11]. Our patient had stenosis of the gastric body and antrum, and SEMS placement was thought appropriate because the length of the stenotic region was 8 to 10cm (a length for which the device might be suitable), the pyloric ring was intact, and some space on the oral side of the stenotic region was available. Finally, stent placement was successfully performed, oral uptake recovered immediately, and our patient’s nutritional status also improved. Stent placement in the upper gastrointestinal tract could be appropriate even for patients with poor performance status and multiple complications because it is less invasive, has a good success rate, and provides rapid symptom relief. Meanwhile, adverse events, including bleeding in less than 1%, pain in 2.5%, perforation in <1%, biliary obstruction in 1.3%, and tumor ingrowth of the stent in 17% to 50%, have been reported [4]. Stent migration also occurred in 0% to 5% with a bare-metal stent (BMS) and in 21% to 26% with a covered stent [4]. Since various complications induced by stent placement often occur in the thoracic upper esophagus within 2cm of the esophageal entrance, esophago-gastric junction, pylorus ring, and ampulla of Vater, the location of stent placement requires careful consideration. Although our patient had stenosis of both the gastric body and the EGJ, with disease progression during first-line chemotherapy, stent placement was used only for the gastric body stenosis, not for the EGJ stenosis. The stenosis of the EGJ was mild and an endoscope could easily pass through the EGJ at that time. In addition, the longest length of WallFlex® Duodenal Stent does not have enough length to cover the distance from the lower esophagus to pre-pylorus. Moreover, stent placement at the EGJ was associated with a risk of stent migration or persistent pain, vomiting and esophagitis due to gastroesophageal reflux, because of expansion of a physically narrowing region. As mentioned above, complications related to stent placement in the EGJ, including dropout of the stent, gastroesophageal reflux, and aspiration pneumonia, have often been reported [12,13]. Recently, an anti-reflux stent that is expected to prevent such complications, has been developed, and its practical effectiveness has been verified [14].

In our patient’s case, stent placement in the gastric body was an effective and durable treatment for the stenosis. Stent placement for stenosis of the esophagus and duodenum has been well documented, but few reports of stenting for gastric body stenosis were found. This might be because carcinomatous stenosis tends to appear in a wide area of the stomach involved with scirrhous type carcinomas. Severe stenosis localized to the gastric body or antrum could be a candidate for this intervention. Selecting appropriate cases and technical improvements could provide greater clinical benefit.

Since the survival benefit of SLC for metastatic gastric carcinoma (MGC) has been proven, it is often considered after failure of the prior chemotherapy [7,8,15]. In recent years, a phase III study showed that SLC with CPT-11 (Camptothecin-11 or Irinotecan) or docetaxel for MGC significantly improved overall survival (OS) compared with best supportive care (BSC) alone (5.1 months for SLC versus 3.8 months for BSC) [15]. SLC with weekly paclitaxel has also been used based on a 16% to 24% tumor response, and overall survival ranged from 3.5 to 8.0 months in several phase II studies [7,8]. In addition, the toxicity of the weekly paclitaxel regimen was generally feasible for patients with MGC, even with moderate carcinomatous ascites [8]. A phase III study comparing bi-weekly CPT-11 (Camptothecin-11 or Irinotecan) and weekly paclitaxel as SLC for MGC has also been conducted in Japan. In our patient’s case, SLC with paclitaxel was safely performed, and it inhibited stenosis of the cardia and re-stenosis of the gastric body and antrum after stent placement, so that long-term oral uptake was achieved. Survival was longer than that reported in the previous report [15]. No mechanical and symptomatic problems were found related to the stent placed in the gastric body and antrum. Severe stenosis of the gastrointestinal tract and fistula formation were often observed in patients with advanced gastrointestinal cancer. These diseases decrease oral uptake and performance status and induce infections such as pneumonia and abscesses. In these cases, it is often difficult to administer anti-cancer agents because of the patients’ poor general condition or the risk of exacerbating infections. However, immediate interventions, such as bypass surgery and stent placement, might make it possible not only to ameliorate symptoms, but also to provide a chance for systemic chemotherapy, resulting in comparative prolongation of survival.

## Conclusions

SEMS placement for carcinomatous stenosis in the gastric body and antrum could be an effective therapeutic strategy for patients in poor general condition in whom bypass surgery is not appropriate. This approach may provide rapid improvement of symptoms, oral ingestion, prolong survival, and allow adequate systemic chemotherapy.

## Consent

Written informed consent was obtained from the patient’s next-of-kin for publication of this case report and any accompanying images. A copy of the written consent is available for review by the Editor-in-Chief of this journal.

## Competing interests

The authors declare that they have no competing interests.

## Authors’ contributions

HK treated our patient and was a major contributor in writing the manuscript. KN, TS, KU and HK were in charge of treatments. TI, MK and ST critically discussed therapeutic plans including endoscopic therapy, chemotherapy and palliative care. RM and EN performed SEMS placement. KA and EB directed our patient’s therapy and were involved in manuscript preparation. All authors read and approved the final manuscript.
